# Blandin's Human and Comparative Dental Anatomy

**Published:** 1845-03

**Authors:** 


*Blandin's Human and Comparative Dental Anatomy.*—We shall commence the publication, in the library part of the June number of our Journal, of a translation, by Robert Arthur, D. D. S., of the above work. It is one of the most popular French works on this department of odontology, that has ever appeared, and if those of our professional friends who do not take the Journal would provide themselves with a copy of the translation, they can have an opportunity of doing so by subscribing for this periodical. By commencing with the fifth volume, they will also secure a copy of the translation of Lefoulon's Dental Surgery.—*Balt. Ed.*

